# Strategies for improving patient recruitment to focus groups in primary care: a case study reflective paper using an analytical framework

**DOI:** 10.1186/1471-2288-9-65

**Published:** 2009-09-22

**Authors:** Jane V Dyas, Tanefa Apekey, Michelle Tilling, A Niroshan Siriwardena

**Affiliations:** 1 National Institute for Health Research, Research Design Service East Midlands, Tower Building, University Park, Nottingham, NG7 2RD, UK; 2 NHS Lincolnshire, Cross O'Cliff Court, Bracebridge Heath, Lincolnshire, LN4 2HN, UK; 3 Faculty of Health, Life & Social Sciences, University of Lincoln Brayford Pool, Lincoln, LN6 7TS, UK

## Abstract

**Background:**

Recruiting to primary care studies is complex. With the current drive to increase numbers of patients involved in primary care studies, we need to know more about successful recruitment approaches. There is limited evidence on recruitment to focus group studies, particularly when no natural grouping exists and where participants do not regularly meet. The aim of this paper is to reflect on recruitment to a focus group study comparing the methods used with existing evidence using a resource for research recruitment, PROSPeR (Planning Recruitment Options: Strategies for Primary Care).

**Methods:**

The focus group formed part of modelling a complex intervention in primary care in the Resources for Effective Sleep Treatment (REST) study. Despite a considered approach at the design stage, there were a number of difficulties with recruitment. The recruitment strategy and subsequent revisions are detailed.

**Results:**

The researchers' modifications to recruitment, justifications and evidence from the literature in support of them are presented. Contrary evidence is used to analyse why some aspects were unsuccessful and evidence is used to suggest improvements. Recruitment to focus group studies should be considered in two distinct phases; getting potential participants to contact the researcher, and converting those contacts into attendance. The difficulty of recruitment in primary care is underemphasised in the literature especially where people do not regularly come together, typified by this case study of patients with sleep problems.

**Conclusion:**

We recommend training GPs and nurses to recruit patients during consultations. Multiple recruitment methods should be employed from the outset and the need to build topic related non-financial incentives into the group meeting should be considered. Recruitment should be monitored regularly with barriers addressed iteratively as a study progresses.

## Background

Timely recruitment is important for completion and generalisability of research studies, but delays and problems recruiting to studies are commonly reported. Campbell et al[1] commented that 53% of studies they reviewed required grant extensions for completion. Barriers to participation are well documented and, although some studies have reported successful recruitment strategies, there is little evidence available to predict the effectiveness of particular approaches[2].

Recruitment to primary care studies is especially complex, because it may involve organisations (general practices or primary care trusts), practitioners (general practitioners, nurses and other primary health care professionals) or patients, in various combinations. Focus groups are an important qualitative method, increasingly being used in primary care as a valuable component in the design and evaluation of complex interventions[3]. Published literature has focused on recruitment to experimental studies and although recruitment to focus groups and other qualitative studies has been previously reported this is usually secondary to the findings of the research itself; due to word count constraints. Discussion of the success or otherwise of recruitment is usually limited and difficulties rarely expanded on. Some researchers have described reasons for non-participation in focus groups and how to overcome these [4-6] but more case studies are required before these findings can be generalised.

With the current emphasis for UK research networks to increase recruitment to studies it is important that existing evidence be utilised in the planning and design of new studies. A practical resource for primary care, PROSPeR (Planning Recruitment Options: Strategies for Primary Care), has been developed from a comprehensive review of evidence using a wide range of published and publicly available sources[7]. A range of recruitment strategies was identified, but with no robust evidence of generalisable effectiveness. The author identified that in primary care there was no single recruitment model that fits all, but that evidence from case studies may be helpful in planning similar studies.

We describe in detail recruitment to a focus group study which was part of a complex intervention study in primary care, Resources for Effective Sleep Treatment (REST). It was selected because, despite a considered approach at the design stage, there were difficulties with recruitment, leading to a revised recruitment strategy. This case study uses PROSPeR to improve our understanding of recruitment to focus groups for future studies in primary care.

## The Case

The aim of the focus group study was to explore experiences of primary care prescribers and patients in the consultation for those presenting with sleep difficulties. More specifically we wanted to discover barriers or facilitators to increasing non-pharmacological interventions and reducing hypnotic prescribing, and to identify aspects of the consultation that contributed to a positive patient experience. Separate focus groups were held with prescribers (general practitioners and nurse prescribers) and patients presenting with insomnia; the recruitment strategy for prescribers was successful, therefore this paper focuses on the strategy for the recruitment of patients.

## Method

### Description of the recruitment approach in the original protocol

#### Patient sample

All general practices in Lincolnshire were invited to express an interest in taking part in the REST project. From 21 practices who expressed an interest, eight were chosen, representing as broad a range of practice characteristics and populations as possible, to form a collaborative in order to pilot and model evidence-based interventions for sleep problems. The collaborative involved regular meetings with the research team at individual practices and together with the other collaborative practices with the main focus being on the development of the interventions. The practices received £2500 over six months to compensate for opportunity costs of practice involvement in the pilot. The requirement for involvement in the focus groups had been discussed at the outset and practices enthusiastically agreed to recruit patients by purposively sampling from their patients presenting with sleep problems in the previous six weeks. The focus groups were a low priority item on the agenda of these meetings.

#### Recruitment and consent procedures

The research team provided patient letters and information sheets to the practices. GPs and nurses were asked to hand out information leaflets and invitation letters to patients who had presented with sleep problems in the previous six weeks and met the inclusion criteria. The invitation letter described the study and asked the patient, if they were interested, to contact the researcher by telephone, or by using a tear-off slip on the letter. The tear-off slip asked the participant to supply a contact telephone number for the researcher to call them. At the initial contact with the researcher further information was given about likely dates and venues, reimbursement of travel expenses, a gift token for taking part and that refreshments were to be provided. To those still interested, the researcher sent out a letter indicating the date, venue and time for the group interview along with the participant information sheet, consent form and initial demographic questionnaire with a stamped addressed envelope. The potential participant was given two weeks in which to consider all the information and to return the completed questionnaire and consent form or bring them along on the day of the focus group, especially if they required the assistance of the researcher in completing them. The researcher's contact details were supplied in case further information was needed at this point. A reminder call was made the day before the group meeting to confirm attendance and remind participants of the venue and time.

#### Inclusion and exclusion criteria

To be included in the study, patients had to have presented with sleep problems during the recruitment period to their GP or nurse prescriber. Patients with a terminal illness or substance misuse who might have specific treatment needs were excluded from the study.

#### Setting

The focus groups took place in private rooms at well known public venues in the vicinity of recruiting surgeries. These included arts centres, libraries and meeting rooms within county town halls. Different times of day were available to accommodate those who worked and those who did not. The venues were usually within travel distance for participants (about 15 minutes to 1.25 hours).

#### Numbers required

A minimum of four focus groups was planned with more if needed to reach data saturation. We aimed for 6-8 participants in each group for ease of facilitation, to give each participant an opportunity to contribute, and in line with recommendations.[8]

This recruitment strategy drew on the combined experiences of the research team with previous focus group studies and extensive reading of the literature (see additional file 1). We considered what might be the most successful way to achieve good recruitment balanced with cost, time available, researcher workload and geographical location.

Tables 1, 2 and 3 are set out in the same way; the first column breaks down into detail the process and the approach to recruiting patients to the focus groups that we tried. The second column gives the reasons why we chose to do things in the way that we did. The last column provides evidence from the literature of why the process or strategy may or may not have worked.

**Table 1:** First modification to the recruitment strategy, including the rationale and evidence for the chosen strategy

Modification to original recruitment strategy	The research team's rationale for the recruitment strategy	Post-project analysis; evidence for the chosen strategy from PROSPeR and other sources
GP to mention study at the end of a consultation, print out invitation letter and give to patient or arrange for the letter to be sent in the post by practice administrators. MODIFIED to In addition to GP/Nurse recruiting during consultation, practice managers will generate a list of patients that have presented with sleeping difficulties in the previous 3 months and practice managers to send out the invitation letters to them on behalf of the GP.	It was not working. It was possible that GPs were acting as gatekeepers. It was possible that GPs/Nurses under the pressure of the consultation were forgetting to discuss the study. It was possible that GPs/Nurses felt that directly mentioning research was intruding on the doctor patient relationship. It was possible that we had just overestimated our recruitment potential.	Studies that required the GP to be alert during consultations were less successful. When GP or practice assistant was the first to inform the patient about the study, patient recruitment was less successful than when the patient received a letter by mail.[65] Not enough time during consultation - impact on working practices. [11,25,66-69] Participation by clinicians in randomised controlled trials was deterred by concern over the doctor-patient relationship.[32] Clinicians who had recruited reported 'trials involve extra work' and 'inviting patients to participate is embarrassing'-these factors affect clinicians' willingness to invite patients to participate.[32] Lasagna's Law (over-optimistic recruitment prediction) holds in Dutch primary care research.[70]
		
Posters displayed in the surgery waiting area inviting patients who have recently consulted their GP with sleeping difficulties to take part in the study by asking them to speak to their GP. **Aspect of recruitment subsequently modified**	We wanted patients to know about the study if it applied to them. To mention it to friends and relatives to whom it might apply. To prepare them to be receptive to the study if the GP discussed it with them. To give patients a chance to be proactive in the recruitment to the study.	With regards to trials in primary care recruitment, a strategy frequently used is waiting room posters informing patients that a study is in progress and targeted poster campaigns to encourage recruitment.[38,57,71,72]

**Table 2:** Second modification to the recruitment strategy, including the rationale and evidence for the chosen strategy

Modification to recruitment strategy 2	Rationale for 2^nd ^recruitment strategy modification	Post-project analysis; evidence for the chosen strategy from PROSPeR and other sources
Posters will be displayed in the surgery waiting area inviting patients who have recently consulted their GP with sleeping difficulties to take part in the study by asking them to speak to their GP MODIFIED to Posters displayed in practices wider than the collaborative and also providing patients with the opportunity to make direct contact with the researcher.	The collaborative funding had ceased, and because the recruitment had not been efficient we wanted a system that necessitated no work on behalf of the practices.	Waiting room posters informing patients that a study is in progress. Targeted poster campaign to encourage recruitment. [38,57,73,74] Provide support from the research team. Invest researcher time and resources to support the study and minimise the impact on the practice.[75]
		
Practice based recruitment was supplemented with an approach that totally removed practice level involvement. We used advertisements in the local news papers covering 7 towns in the county. The advert invited patients experiencing sleeping difficulties during the previous 6 months to contact the researcher.	There had already been local news coverage countywide about the study, so we hoped that an invitation using the study logo might appeal to people who had noticed the first publicity. The county is rural and the papers cover areas wider than the collaborative practices. It might appeal to a wider group of people.	Provide support from the research team. Invest researcher time and resources to support the study and minimise the impact on the practice.[75] Repeated publicity at a local level delivered by locally based investigators well known to their primary care community.[76]

**Table 3:** Recruitment approach, time taken to achieve recruitment, recruitment achieved

Recruitment strategy	Time taken to achieve recruitment (weeks)	Focus Group Venues	Number of individual interviews held at focus group venue	Number contacting researcher	Number agreeing to attend on the day before the group meeting	Number attending and being interviewed
**Strategy 1**		Bourne	0	0	0	0
	7	Grantham	0	2	0	0
	13	Lincoln	0	7	6	3
		Louth	0	0	0	0
**Strategy 2**	4	Bourne	0	1	0	0
	7	Grantham	1	3	3	1
	6	Lincoln	0	6	4	0
	7	Louth	1	5	5	1
**Strategy 3**	1	Boston	0	6	4	4
	2	Bourne	0	3	3	2
	2	Grantham	0	4	4	3
	1	Lincoln 1	0	5	5	5
	1	Lincoln 2	0	4	4	4
	1	Louth	1	1	1	1
	1	Sleaford	1	1	1	1
	1	Skegness	0	3	2	2

#### Ethics approval

Ethical approval was obtained from the North Nottinghamshire Research Ethics Committee. Ethics number 07/H0407/59.

## Results

### Modification 1 to the recruitment strategy

The rate at which potential participants contacted the researcher was initially very slow. Discussion with recruiting GPs revealed that some were acting as gatekeepers, only inviting patients they thought would be articulate or who they classed as having a serious sleep problem, rather than following the inclusion criteria of inviting anyone who raised difficulty sleeping during the consultation. During this period we provided feedback on the actual recruitment rates to the collaborative practices at their group meetings and during practice visits and urged practices to continue to recruit, but this did not improve participation. We therefore had to modify our approach. In addition to GPs and nurses identifying patients during the consultation, lists were generated by the practice managers of all patients who had sleep difficulties using clinical (Read) codes for insomnia and hypnotic drugs. They then arranged for invitation letters to be sent out on behalf of the practice. The inclusion criterion was changed from those presenting with sleep difficulties during the previous 6 weeks to the previous 3 months. These modifications (Table 1) were classed as substantial modifications and required further ethics committee approval.

### Modification 2 to the recruitment strategy

Because uptake was still low we made a second modification to our recruitment strategy (Table 2). During this time we individually interviewed several patients who had been unable to attend a focus group for personal diary reasons. Data saturation had not been achieved although the information we were collecting was rich and informative. We continued to recruit through the practices but decided to supplement this with an approach that removed practice involvement using advertisements in local newspapers covering seven towns in the county. The advert invited patients experiencing sleeping difficulties and who had contacted a GP during the previous six months, to contact the researcher. The poster was modified to include the researcher contact details in addition to encouraging patients to speak to their GP. This poster was also sent by email to all general practices within the seven towns asking them to display it in their reception area. Patients who contacted the researcher remained anonymous, until, as in the original protocol they themselves gave the researcher their contact details. Screening questions to establish eligibility for inclusion were also asked whilst the caller was still anonymous for all patients that contacted the researcher. Once it had been established that the caller was eligible, recruitment proceeded as in the original protocol. This modification required further ethics approval. The modifications led to increasingly successful recruitment to the focus groups sufficient to complete recruitment to the study (Table 3). This table provides the raw data showing how modifications to the research strategy speeded up the rate at which patients came forward, and the final numbers being interviewed, either individually or in focus groups.

The modifications that we made to our recruitment strategy required further ethical consideration by the Ethics Committee and therefore we had to re-submit information to them on several occasions. This information is summarised in table 4.

**Table 4:** Timeline for the modification made to the recruitment and consent procedures

Original recruitment and consent procedures and subsequent modifications	Date
Ethics meeting	03/09/07
Letter from ethics asking for more information	17/09/07
Letter of approval from ethics	14/11/07
Amendment 1 submitted	07/01/08
Amendment 1 approved	07/02/08
Amendment 2 submitted	17/04/08
Amendment 2 approved	16/05/08
Study officially ended	01/09/08

## Discussion

### Summary of main findings

The difficulty of recruiting patients to focus groups in primary care is underemphasised in the literature[9]. This is particularly true of studies where there is no natural grouping and where people do not regularly come together, typified by this case study of patients with sleep problems. Our initial strategy contended with the wide geographical spread of practices, which meant that patients, unable to attend on arranged dates, could not easily join focus groups taking place in another town.

The recruitment process we used had two distinct phases, the first was getting potential participants to contact the researcher, and the second was converting those contacts into attendees at the venues through the information giving, consenting and facilitating processes that were the responsibility of the researcher. We needed two modifications to the initial recruitment process to achieve data saturation.

### Comparison with existing literature

Altogether, of 51 people that contacted the researcher, 42 (82%) agreed to take part and of these 42, 27 (64%) gave data at the arranged venues; so 53% of those contacting the researcher actually took part in the research. By comparison, 22 (14%) of 160 initial responders contributed data to a focus group study conducted by Richards et al [10]. Therefore our main revision needed to concentrate on phase one, reaching potential participants and getting them to contact the researcher. There was little evidence in the literature to suggest that the personalised approach to follow up by the researcher with appropriate incentives was not the best way forward.

The timeliness and importance of the subject under study may have been a key factor influencing our low phase one recruitment rate[7]. People only attend focus groups if they think the topic is important[8] and have a personal interest in it[11].

Our original recruitment strategy was founded on our belief that this subject was of sufficient interest to engage practitioners and patients of practices that were involved. This was based on our previous response to questionnaire studies of views of patients and practitioners on insomnia[12,13].

However, for patients, responding to a questionnaire in the comfort of ones home is very different from putting yourself out to attend a group discussion, particularly when personal aspects may be discussed. The degree of importance attached to taking part in the research has to be high to outweigh conflicts with domestic, lifestyle, or work commitments [14-18]. Recruitment to focus groups has been previously shown to be difficult when taking part in research is not seen as a priority for potential participants[19]. Furthermore, although insomnia is common[20] and disruptive to individuals' lives we found from the focus groups that many patients manage to function and do not perceive sleep difficulties as a medical condition[21]. These factors may have affected willingness to express any interest in taking part in the research explaining our low phase one recruitment.

If patients do not take up invitations to take part in focus groups due to the importance of the topic, can they be persuaded to attend through incentives? Financial or other incentives cannot be advertised to attract patients because of potential selection bias. Hoddinott advises assessing which non-financial incentives may matter most to potential participants[22] such as individual learning, contribution to medical knowledge, improved future patient care or just giving something back[23].

Such incentives will be linked to the patient's view of importance. In our example altruism was insufficient to attract the initial expressions of interest in participating. However the importance of taking part in the group might have been improved by offering an alternative non-financial incentive such as a sleep hygiene session by a nurse to attendees at the end of the meeting.

With regards to the known difficulties in asking GP's and nurses to recruit patients in the routine consultation, our main design fault was in assuming that the financial incentives, together with the GP's personal interest in insomnia and their working relationship with the lead researcher would be sufficient motivation to outweigh difficulties such as lack of time[24]. Concerns about effect on the doctor-patient relationship or the perceived appropriateness of raising research during sensitive consultations, may also have played a part in the low phase one recruitment [25-28].

There were three main aspects to our original recruitment strategy that can be improved in future studies. The first is not to assume that being interested in a subject will equate to it being given importance with regards to recruitment to the focus groups. This is supported by the fact that as soon as we started to recruit using newspaper advertisements the practices did not recruit any further patients. The low importance given to recruiting patients and the poor involvement of practices with the process may have resulted from insufficient attention to engaging practices and marketing the focus groups[7]. That is, we only discussed the focus groups within the context of the collaborative meetings where the piloting of the insomnia interventions was the main focus of discussion. We could have had a separate meeting to prepare practices for the focus group recruitment, as it is known that GPs need a clear description of what is required of the practice or personnel[29]. Furthermore we could have placed greater emphasis on individualising approaches to practices, meeting with them separately to discuss specific organisational issues rather than our collective approach[30].

The second lies in not assuming that financial incentives are important motivators to general practices when there is no firm evidence that payment to health care professionals improves recruitment[7]. Despite extensive use of payments to healthcare professionals for patient recruitment to trials, a systematic review showed that payments had limited effectiveness in improving both quantity and quality of recruitment[31].

Finally, it is important to recognise that although clinicians do sign up to projects, especially when they know the researcher, this does not always translate into sufficient motivation to recruit[32]. The fact that some practitioners were not adhering to the inclusion criteria suggests that we had not spent sufficient effort in engaging GPs to follow the protocol[33].

These findings offer possible explanations for slow phase one recruitment to our study despite modifications to the process. However, persistence, changing approaches and making modifications based on rationale and evidence, resulted in sufficient data saturation for our study. The benefits of a flexible recruitment protocol[34,35] using multiple methods[36] is supported as a tactic for recruiting patients to primary care studies.

## Conclusion

From our analysis of evidence from the literature and this case study we make a number of general recommendations for improving recruitment to focus groups where the research question requires a broad spectrum of views to be collected from people who would not normally gather together for the topic of focus.

1. Ensure good engagement of clinicians (GPs and nurses). In future we might try to achieve this by providing special education and training on how to introduce research during a consultation[37] or running workshops for recruiters[38].

2. Design the focus group study with scope for multiple recruitment methods at the outset to avoid delays in returning for ethical approval[39].

3. Consider the need to build topic related non-financial incentives into the focus group meeting itself as a means of raising the importance of attending group discussion with patients.

Finally research teams should regularly monitor recruitment to focus groups and be prepared to address barriers as they go along, using an iterative approach in order to achieve satisfactory levels of participation[34,40,41]. It remains the case that these are suggested strategies only and it would therefore be useful if any future studies adopting such approaches could report on their effectiveness in the growing literature on recruitment.

Finally with regards to the usefulness of the PROSPeR resource; a future study, using it to plan a recruitment strategy, followed by an evaluation is recommended. We also recommend it as a diagnostic tool when researchers meet recruitment and retention difficulties with primary care studies.

## Competing interests

Jane Dyas is a member of the Trent RDSU which publishes PROSPeR on their website http://www.trentrdsu.org.uk/resources_recruitment.html and is a member of the project steering group.

## Authors' contributions

JD was responsible for the design of the study, data collection, data analysis and the writing of the first draft of the paper. TA carried out recruitment throughout the study and was involved in the recruitment design and data analysis. MT was involved in the recruitment design and was responsible for managing recruitment. NS was involved in the design of the study and revisions to the paper. All authors read and approved the final manuscript.

## Pre-publication history

The pre-publication history for this paper can be accessed here:

http://www.biomedcentral.com/1471-2288/9/65/prepub

## Supplementary Material

Additional file 1 **Rationale for original recruitment strategy and a post project analysis of the recruitment strategy**. The table provides information about the original recruitment strategy and a post-project analysis. Click here for file
