# Supplementary material for: Strategies for improving patient recruitment to focus groups in primary care: a case study reflective paper using an analytical framework
Source: BMC Med Res Methodol. 2009 Sep 22;9:65. doi: 10.1186/1471-2288-9-65 (PMC2759948; doi:10.1186/1471-2288-9-65)
Supplement: Additional file 1 — Rationale for original recruitment strategy and a post project analysis of the recruitment strategy. The table provides information about the original recruitment strategy and a post-project analysis. [file 1471-2288-9-65-S1.DOC]

Rationale for original recruitment strategy and a post project analysis of the recruitment strategy

| **Aspect of original recruitment strategy** | **The research team’s rationale for the recruitment strategy** | **Post – project analysis; evidence for the chosen strategy taken from PROSPeR and other sources** |
| --- | --- | --- |
| A service user representative attended the first meeting to design the study and was included in subsequent consultations regarding the protocol. Recruitment and patient information was particularly focused on the lay opinion. | We needed to know how a patient might prefer to be asked to join a focus group study to discuss their consultation on sleeping difficulties. The user opinion was that patients would take the research more seriously if the invitation came from his/her GP. | The development of the research question and study design should include consultation and collaboration with clinicians and participants.[11, 25, 42-45] |
| Researcher to prepare all paperwork including initial invite letters, and to handle all aspects of the recruitment other than sending out the initial invite. | GPs would not have time to be involved in anything but a minimal input. | Time pressures are often cited as a reason for non participation by practitioners and their staff therefore investigators should transfer as much of the study burden from participating physicians to project staff as possible .[46]  The research team should minimise the workload of those involved in recruitment. Recruitment and informed consent undertaken by a researcher.[29, 47, 48] |
| GP’s to be briefed about the focus group study at collaborative group meetings. This included explaining the inclusion and exclusion criteria.  Further details were provided by the researcher and project manager on their practice visits. | Our aim was to make the focus group study part of the collaborative group agenda, because we anticipated that doing so would save practitioner travel time and have the added value of enabling the whole group to ask relevant questions. | There is a clear relationship between the complexity of the inclusion procedures and success of recruitment.[49] |
| GP to mention study at the end of a consultation, print out invitation letter and give to patient or arrange for the letter to be sent in the post by practice administrators.  **Aspect of recruitment subsequently modified** | The lead researcher was a GP who had good pre-existing relationships with the practices and considered the approach would work  The GP practices (primary care organisation) had already stepped forward as enthusiasts for the topic described by the lead researcher as “a group of committed clinicians”.  GPs in the collaborative had been asked if they were willing to recruit; they had agreed and made no comments that the system would be unworkable or the numbers likely to be insufficient.  GP practices had been paid to be involved in the study, which included recruiting to focus groups. | Physician personal contact and friendship networks are powerful tools for recruitment.[50] Lasagna’s Law (over-optimistic recruitment prediction) holds in Dutch primary care research.[51]  For recruiting primary care practitioners a number of papers point out that the priority and level of interest in the topic and the relevance of the research question are important. [11, 34, 52-57]  GPs already taking part in an existing research network were more willing to participate than others.[58]  Reimbursement of excess costs associated with recruiting patients and taking part in research.[29]  Direct personal approach more likely to result in successful recruitment.[59] |
